# You Are What You Eat: A Genomic Analysis of the Gut Microbiome of Captive and Wild *Octopus vulgaris* Paralarvae and Their Zooplankton Prey

**DOI:** 10.3389/fphys.2017.00362

**Published:** 2017-05-31

**Authors:** Álvaro Roura, Stephen R. Doyle, Manuel Nande, Jan M. Strugnell

**Affiliations:** ^1^Department of Ecology, Environment and Evolution, La Trobe UniversityMelbourne, VIC, Australia; ^2^Ecología y Biodiversidad Marina, Instituto de Investigaciones Marinas (CSIC)Vigo, Spain; ^3^Parasite Genomic Group, Wellcome Trust Sanger InstituteCambridge, United Kingdom; ^4^Grupo de Acuicultura Marina, Instituto Español de OceanografíaVigo, Spain; ^5^Departamento de Bioquímica, Genética e Inmunología, Universidad de VigoVigo, Spain; ^6^Marine Biology and Aquaculture, James Cook UniversityTownsville, QLD, Australia

**Keywords:** *Octopus vulgaris* paralarvae, high throughput sequencing, gastrointestinal tract, microbial communities, core gut microflora, upwelling ecosystems, aquaculture microbiology

## Abstract

The common octopus (*Octopus vulgaris*) is an attractive species for aquaculture, however, several challenges inhibit sustainable commercial production. Little is known about the early paralarval stages in the wild, including diet and intestinal microbiota, which likely play a significant role in development and vitality of this important life stage. High throughput sequencing was used to characterize the gastrointestinal microbiome of wild *O. vulgaris* paralarvae collected from two different upwelling regions off the coast of North West Spain (*n* = 41) and Morocco (*n* = 35). These were compared to that of paralarvae reared with *Artemia* for up to 25 days in captivity (*n* = 29). In addition, the gastrointestinal microbiome of zooplankton prey (crabs, copepod and krill) was also analyzed to determine if the microbial communities present in wild paralarvae are derived from their diet. Paralarvae reared in captivity with *Artemia* showed a depletion of bacterial diversity, particularly after day 5, when almost half the bacterial species present on day 0 were lost and two bacterial families (Mycoplasmataceae and Vibrionaceae) dominated the microbial community. In contrast, bacterial diversity increased in wild paralarvae as they developed in the oceanic realm of both upwelling systems, likely due to the exposure of new bacterial communities via ingestion of a wide diversity of prey. Remarkably, the bacterial diversity of recently hatched paralarvae in captivity was similar to that of wild paralarvae and zooplankton, thus suggesting a marked effect of the diet in both the microbial community species diversity and evenness. This study provides a comprehensive overview of the bacterial communities inhabiting the gastrointestinal tract of *O. vulgaris* paralarvae, and reveals new research lines to challenge the current bottlenecks preventing sustainable octopus aquaculture.

## Introduction

One of the most outstanding issues in microbial ecology of the gastrointestinal (GI) tract is understanding how biological and physical factors influence gut microbiota and their hosts (Sullam et al., 2012). The GI tract is occupied by a complex and dynamic ecosystem of organisms composed of an enormous variety of aerobic, facultative anaerobic and obligate anaerobic microbes that interact with the host and with each other (Nayak, 2010). The role of microbiota on host health is increasingly being recognized, for example, the GI microbiota of fish contributes to the development of its host through xenobiotic metabolism, microbially-mediated digestion of food, essential nutrient supply including vitamins, amino acids and fatty acids, immunity, and resistance toward intestinal pathogens (Kesarcodi-Watson et al., 2008; Ringø et al., 2016).

Until recently, most studies examining the microbiota associated with marine organisms have employed culture-dependent methods (Forney et al., 2004). This approach is somewhat limited, given that the vast majority of microorganisms present in a natural environment cannot be cultured *in vitro* (Fjellheim et al., 2007). For example, in marine biomes the percentage of unculturable organisms is estimated to be higher than 97% (Rappé and Giovannoni, 2003). Culture-independent methods, such as the detection and sequencing of the microbial-derived 16S small subunit ribosomal RNA (rRNA) gene, have been developed to overcome this limitation, and have been applied toward the study of hatchery-associated bacterial populations of Atlantic cod (*Gadus morhua*, Brunvold et al., 2007; Reid et al., 2009; Bakke et al., 2013), abalone (*Haliotis diversicolor*, Zhao et al., 2012), Atlantic halibut (*Hippoglossus hippoglossus* L., Verner-Jeffreys et al., 2003; Jensen et al., 2004) and great scallop (*Pecten maximus*, Sandaa et al., 2003). However, the focus on PCR amplification of the 16S rRNA gene alone may provide a biased estimate of species abundance, given that “universal primers” for 16S PCR are not necessarily universal, i.e., not all species can be detected due to unknown sequence variation, and that biases associated with primer mismatch, and preferential amplification of the most abundant groups have been described (reviewed in Forney et al., 2004).

High throughput sequencing (HTS) methods are increasingly being applied to the characterization of microbial communities, leading to a more comprehensive appreciation of extant biodiversity (Mock and Kirkham, 2012). Although, HTS approaches still commonly rely on the amplification of one or two hypervariable regions of the 9 hypervariable regions (V1–V9) present in the 16S rRNA gene, its significant advantage over conventional 16S rRNA sequencing is the dense sampling of a given community increasing the likelihood of capturing lowly abundant species (Mock and Kirkham, 2012). Moreover, the sample throughput of HTS is significantly higher than traditional approaches, mediated by sample barcoding and multiplexing (the number of samples limited largely by the number of unique barcodes available), enabling study designs thousands of times more robust than other PCR-based techniques (Zarkasi et al., 2014; Huang et al., 2016).

The efficiency and sustainability of any mariculture system will likely be significantly influenced by the microbial composition of the species in question in their natural environment. Characterization of species composition, relative quantities, and the potential sources of the core intestinal microbiota commonly associated with feed and larvae at different stages of development is essential for viability and vitality of the organisms (Ringø and Birkbeck, 1999; Olafsen, 2001), and aid in the identification of possible microbial pathogens affecting larval mortality (Star et al., 2013). In cephalopod mollusks (octopus, squids and cuttlefishes), only three studies have analyzed the microbial diversity of *Octopus* species (de la Cruz-Leyva et al., 2011; Iehata et al., 2015, 2016). These studies have used Denaturing Gradient Gel Electrophoresis (DGGE) techniques to analyse cultured bacterial diversity sampled from adults and eggs of the Chilean Gould octopus, *Octopus mimus* (Gould, 1852), revealing differences between males and females in the microbial families present (mostly Vibrionaceae and Streptococcaceae) and their nutritional enzymatic activities (Iehata et al., 2015). In addition, a relationship between egg-associated bacterial diversity and egg health condition (dominated by *Roseobacter*) was also detected (Iehata et al., 2016). Vibrionaceae were the main bacterial group, identified using RNA transcripts of the 16S rRNA gene, from metabolically active bacterial flora of adult octopuses collected in Mexico (de la Cruz-Leyva et al., 2011). Considering the low throughput approaches used in these studies, it is likely that they only account for a fraction of bacterial diversity present within octopods or cephalopods in general. The application of genomic methods will significantly enhance the characterization of the cephalopod paralarvae microbial communities, and may in turn provide useful insight toward improving the aquaculture conditions of commercially important species such as the common octopus, *Octopus vulgaris* Cuvier, 1797.

In spite of the plethora of experiments to solve it (reviewed in Vidal et al., 2014), rearing *O. vulgaris* paralarvae in captivity is difficult and remains a significant hurdle that prevents viable aquaculture. Little is known about the ecology of wild *O. vulgaris* paralarvae and their unusual planktonic strategy in the open ocean, largely due to difficulties in obtaining specimens (Roura, 2013). It has been recently suggested that *O. vulgaris* paralarvae undertake a unique planktonic strategy, compared with that of other coastal cephalopods with planktonic stages (Roura et al., 2016). They hatch close to the coast with only three suckers per arm, and after <10–15 days, they are transported away from the continental shelf by coastal upwelling filaments, finishing their development in the open ocean. Remarkably, 58 *O. vulgaris* paralarvae containing more than three suckers per arm were collected in zooplankton samples off the NW Iberian Peninsula (42 specimens) and Morocco (16 specimens), with bottom depths ranging between 787 and 3,110 m (Roura, 2013). These paralarvae are the only specimens larger than three suckers per arm ever collected in the Eastern Atlantic (Rocha et al., 1999; González et al., 2005; Moreno et al., 2009; Otero et al., 2009; Roura et al., 2016). These rare samples therefore provide a unique opportunity to study the ontogenic changes of their microbial biota from the coast to the ocean, and to compare the natural microbiome against that found in aquaculture, with the aim to determine the importance of the GI microbiome on the health of captive paralarvae.

In this study, we have applied HTS to characterize the core gut microbiota of wild paralarvae collected in two different upwelling regions (NW Spain and W Morocco), and to identify the main microbial groups that differ between ecosystems. Furthermore, we have compared the GI microbiota of wild *Octopus* paralarvae against that of paralarvae reared with *Artemia* during 25 days in captivity. This enabled characterisation of the core gut microbiota of wild paralarvae and identification of bacterial groups that are not present in paralarvae reared in captivity, and to identify potential pathogens that may affect the health of paralarvae reared in captivity.

## Materials and methods

**Figure 1:**
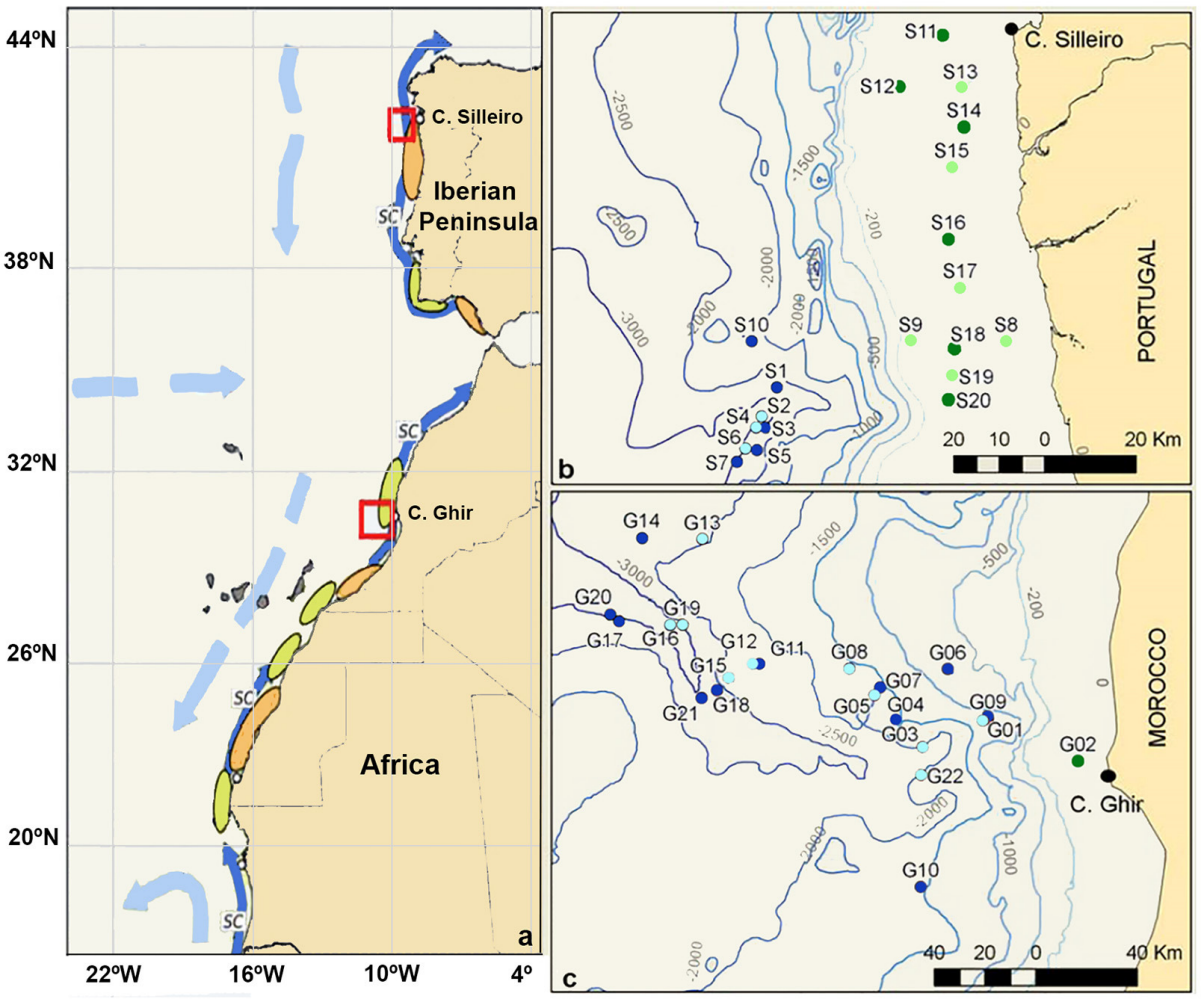
**(a)** Schematic map of the Iberian Canary Current eastern boundary upwelling showing the areas sampled (red boxes) and the main currents (light blue: surface currents; dark blue: slope current = SC), retention (orange), and dispersion (green) zones on the shelf. **(b)** Zooplankton samples collected off the coast of the NW Iberian Peninsula. **(c)** Zooplankton samples collected off the Morocco coast. Samples collected over the continental shelf (green, <200 m depth) and in the open ocean (blue, >200 m depth), with light/dark colors representing day/night samplings.

Planktonic samples were collected during the multidisciplinary project “Canaries-Iberian Marine Ecosystem Exchanges (CAIBEX)” (Figure 1, red frames), off the coasts of North-Western Iberian Peninsula (CAIBEX-I: July 7 to 24, Figure 1B) and Morocco (CAIBEX-III: August 16 to September 5, Figure 1C) in 2009. Mesozooplankton samples were collected day and night with two 750 mm diameter bongo nets equipped with 375 μm mesh and a mechanical flow-meter. Three double-oblique towings were carried out (at a ship speed of 2.5 knots) per station over the continental slope (>200m depth): (i) at the deep scattering layer (DSL: 500 m), (ii) at 100 m, and (iii) at the surface (0–5 m). Over the continental shelf (<200 m) only two double oblique towings were collected at 100 m (when sea-bottom was <100 m, otherwise 10 m above it) and at the surface (0–5 m). The bongo net was first lowered to the desired depth, towed for 30 min and subsequently hauled at 0.5 m s–1. The net was recovered, cleaned on board and placed back into the sea for the next towing. Plankton samples were fixed with 96% ethanol and stored at −20°C to facilitate DNA preservation. All cephalopod paralarvae were sorted from the zooplankton samples and stored individually in 70% ethanol at −20°C. In total, 134 *O. vulgaris* paralarvae were collected during CAIBEX-I (*n* = 99 specimens) and CAIBEX-III (*n* = 35 specimens). Of these, 41 paralarvae were chosen from CAIBEX-I (ranging from 3 to 5 suckers per arm) and 35 from CAIBEX-III (ranging from 3 to 15 suckers per arm) to study the ontogenic changes of the microbiota in the wild.

The microbiota of paralarvae reared in captivity with *Artemia* in 2012 at the facilities of the Spanish Institute of Oceanography in Vigo (IEO-Vigo), was also analyzed using five replicates at ages 0, 5, 10, 15, 20, and 25 days post hatchling (ranging from 3 to 5 suckers per arm). Paralarvae were anesthetized at the end of the study by immersing them in a 1.5% MgCl_2_dissolved in seawater at room temperature (18–21°C) for 10 min, after which the MgCl_2_ concentration was increased to 3.5% for 30 min to kill them. The procedures applied herein comply with Directive 2010/63/EU, in terms of minimizing the number of animals used and animal sacrificing method employed (Fiorito et al., 2015). This study was performed in accordance with corresponding Spanish guidelines and regulations (Ley 32/2007, November 7th) and was exempt from an ethics review process.

The euphausiid *Nyctiphanes couchii*, the crabs *Pirimela denticulata* and *Pilumnus hirtellus*, and the copepod *Paraeuchaeta hebes*, were sorted from the zooplankton samples collected near the coast of NW Spain and the microbiome of their gastrointestinal tract was analyzed. Crabs and krill are known prey of wild *O. vulgaris* paralarvae (Roura et al., 2012), whereas *P. hebes* has not been described as part of the Octopus diet. However, this copepod is an important member of the coastal zooplankton (Roura et al., 2013) and has been recently identified in the digestive tract of *Alloteuthis media* paralarvae (Olmos-Pérez et al., 2017), and therefore, has been included as a potentially informative bioindicator of the environmental microbiota present.

### Library preparation and sequencing

Genomic DNA was extracted from the dissected digestive tract of *O. vulgaris* paralarvae (including the esophagus, crop, stomach, caecum, digestive gland, and intestine) and zooplankton prey (including the internal contents of the cephalothorax after removing appendages and the carapace). DNA was extracted with QIAGEN DNeasy Blood and Tissue Kit according to manufacturer's instructions. A slight modification was made at the final elution stage; the elution was repeated twice using two 20 μL aliquots of 45°C ultrapure water, and stored as a combined 40 μL eluate prior to use.

A DNA fragment that spanned the V3 and V4 hypervariable regions of 16S rRNA (~444 bp) was amplified with the primers S-D-Bact-0341-b-S-17 (341f)/S-D-Bact-0785-a-A-21(785r; Klindworth et al., 2013), since it is the optimal hypervariable regions to characterize bacterial communities (Mizrahi-Man et al., 2013; Sinclair et al., 2015). These primers included a modification to the 5′ end to include an Illumina-compatible adapter sequence to allow multiplexing (Table 1 in bold). An evaluation of base-specific biases for the commonly used PCR primer sets used to amplify the 16S rRNA hypervariable regions compared with metagenomic data, revealed that <16% of 16S rRNA sequences are missed with the V3–V4 regions (Eloe-Fadrosh et al., 2016). They defined a subset of bases within the “universal” primers contributing to the percentage of metagenomic SSU rRNA gene sequences that would probably be missed in next generation PCR-based surveys. Accordingly, we modified one of these variable nucleotides by adding an inosine (I) to complement all four nucleotides (Geller et al., 2013) in the 3′ end of the universal primer 341f (Table 1 in italics) to capture a greater fraction of the microbial diversity.

**Table 1:** Primers used in this study, modified from Klindworth et al. (2013) to include Illumina adapter overhang nucleotide sequences (in bold) and an inosine (I) in the 3′end of 341f primer instead of *N* (i.e., A or T or C or G).

**Primer**	**Sequence 5′–3′**
341fI	**TCGTCGGCAGCGTCAGATGTGTATAAGAGACAG** CCTACGGG*I*GGCWGCAG
785r	**GTCTCGTGGGCTCGGAGATGTGTATAAGAGACAG** GACTACHVGGGTATCTAATCC

PCR reactions contained 0.35 μl of primer 341f and 0.2 μl of primer 785r (10 μM stock concentration), 6.25 μL REDTaq® ReadyMix (Sigma-Aldrich), 0.1 μL MgCl_2_ and 1 μL of DNA (at a concentration of ~20 ng) in a total reaction volume of 12.5 μL. Touchdown PCR cycle conditions included an initial denaturing step (95°C for 3 min), followed by 10 cycles at 95°C for 30 s, 58°C for 30 s (1° decrease per cycle) and 72°C for 30 s; followed by 15 cycles at 94 °C for 30 s, 48°C for 30 s, and 72°C for 30 s. Negative control reactions containing all components, but water instead of template, were performed alongside all PCR reactions to ensure that there was no contamination.

PCR products (2 μl) were visualized on a 1% (w/v) agarose gel. Five microliters of this PCR product was added to a second PCR reaction for 10 cycles (95°C for 10 s, 48°C for 15 s, and 72°C for 15 s), in order to incorporate Illumina dual index primers (4 μL of 1.25 μM) to the V3–V4 amplicon target by re-amplification. Amplified DNA solutions were purified using AMPure XP beads/PEG 6000 solution (1.1 × beads/DNA volume), quantified using a Qubit® 2.0 Fluorometer (Invitrogen) and pooled in equimolar concentrations (0.5 ng/μL). The library was diluted to 12.5 pM and sequencing was performed using a 600 cycle (paired-end) v3 MiSeq Reagent Kit on an Illumina MiSeq. PhiX sequencing library (Illumina) was spiked into the amplicon sequencing library (10%), to account for the limited sequence diversity among the 16S amplicons.

### Quality filtering and bioinformatic analysis

Quality filtering was carried out following recommendations for Illumina platforms (Bokulich et al., 2013). Reads that did not meet the following standards were removed: (i) Phred score below 30 (i.e., one error in 1,000 bases), (ii) less than 75% of target length, (iii) less than three consecutive low quality calls, and (iv) reads with ambiguous calls. The remaining paired-end reads were merged using PEAR v0.9.4 (Zhang et al., 2014). Merged reads were demultiplexed into individual sample read-sets based on their corresponding indexed adapter combination. Reads for which the indexes/primers did not match the expected sequences were discarded. The remaining reads were then filtered against a custom Kraken (v0.10.4) database to exclude archaeal and viral contamination (Wood and Salzberg, 2014). The UCHIME algorithm of USEARCH (v 6.0.307; Edgar et al., 2011) was used to check for chimeric sequences amongst the bacterial reads.

Bacterial reads were then classified using ClassifyReads, a high-performance naïve Bayesian classifier of the Ribosomal Database Project (RDP) described in Wang et al. (2007) available within the Illumina metagenomic analysis software 16S Metagenomics on BaseSpace (https://basespace.illumina.com). ClassifyReads uses a 32-character kmer word-matching strategy to determine the percentage of shared words between a query and the Greengenes taxonomy database (greengenes.secondgenome.com/downloads). This database is currently based on a *de novo* phylogenetic tree of 408,135 quality-filtered complete sequences calculated using FastTree (McDonald et al., 2012). Taxonomy was assigned to each read by accepting the Greengenes taxonomy string of the best matching Greengenes sequence (127,741 complete bacterial sequences; Werner et al., 2012). We selected this classification method due to favorable trade-offs among automation, speed, and taxonomic accuracy (Liu et al., 2008; Werner et al., 2012).

The RDP classifier uses a bootstrapping method of randomly subsampling the words in the sequence to determine the classification confidence (Wang et al., 2007). However, the error rate associated with a confidence threshold is dependent on several factors, including the taxonomic resolution of the prediction (kingdom vs. genus), the sequence length for classification, and the amplified region of the 16S rRNA gene. Consequently, the use of one overall “confidence” threshold for classification, for example 80% (Wang et al., 2007) or 50% (Claesson et al., 2009), often results in sub-optimal and unequal performance across regions and taxonomic ranks (Mizrahi-Man et al., 2013). In ClassifyReads, there is no bootstrapping procedure and confidence is statistically assigned based on the overall accuracy of the classification algorithm at different taxonomic levels (ranging from 100 to 98.24%, from kingdom to species). Reads that did not match a reference sequence were considered as unclassified and were included in the community analysis, since they represent an important source of bacteria particularly in anaerobic systems (Werner et al., 2012).

### Multivariate analysis of microbial communities

Relative abundances were calculated using the Greengenes classifications of the OTUs. Microbial community structure was examined with multivariate techniques using the software package PRIMER6 & PERMANOVA+ (Anderson et al., 2008). Genus relative abundances for all samples were log transformed (x + 1) to improve homogeneity of variance, and a Bray-Curtis similarity matrix was generated. A principal coordinate analysis (PCO) ordination was used to visualize the natural groupings of the samples using 2D and 3D plots. The natural groupings emerging from the PCO plot were further analyzed with PERMDISP, based on distances to centroids, to examine the dispersion among groups (Anderson, 2004). Subsequently, a non-parametric permutational ANOVA (PERMANOVA) analysis was used to test for statistical differences in the multidimensional space. PERMANOVAs were based on the Type III (partial) sum of squares and 999 permutations of residuals under a reduced model.

Relationships between the resemblance matrix of microbial families and explicative variables were explored with distance-based linear models (DistLM). We grouped the different variables in four sets: (i) *Run*: reads passing filter, reads classified, dilution/addition (2 categories); (ii) *Taxonomy*: bacterial, archaeal and viral reads, Shannon's species diversity index (H′), taxa identified (phyla, class, order, family, genus and species); (iii) *Experiment*: origin of samples (4 categories: Morocco, NW Spain, Aquaculture and zooplankton), day/night (categorical), strata (3 categories: 5, 100, and 500 m), coast/ocean (categorical); and (iv) *Octopus*: captive/wild (categorical), sucker number, dorsal mantle (DML), total length (TL), width, distance to coast, depth and age. Prior to modeling, all variables were tested for collinearity (Spearman correlation matrix) and those with determination coefficients (*R*^2^) higher than 0.9 were omitted. The retained variables were then transformed to compensate for skewness when needed applying log (x + 1).

The contribution of these four sets of variables to the total variability found in the microbial resemblance matrix was determined using a step-wise selection procedure using the adjusted *R*^2^ as selection criterion. All significant variables were introduced in the model with the “best” procedure of the DistLM model using the Bayesian information criterion (BIC), as it includes a more severe penalty for the inclusion of new predictor variables than Akaike's information criterion (AIC). Such a procedure permitted developing the simplest model to explain the microbial community structure. The output of the fitted model was visualized with distance-based redundancy analysis (dbRDA; Anderson et al., 2008).

The microbial families contributing most to similarities and dissimilarities among wild and captive paralarvae and the zooplankton were determined using the program SIMPER (Anderson et al., 2008). This analysis allowed recognizing the core gut microflora of wild and captive paralarvae, their contribution to the total community, and the discriminative power of the main families driving the differences between communities.

## Results

### *Octopus* samples

The 105 wild *Octopus* paralarvae analyzed in this study ranged from 1.30 to 5.01 mm in dorsal mantle length, contained 3–15 suckers per arm and were captured between 10 and 171 km off the coast (see more details on Table 2). The paralarvae found in the open ocean were thoroughly sampled in both upwelling systems, because they are essential to understand the ontogenic changes of the GI microbial communities during the transition from the coastal hatchling grounds (*n* = 19) to the oceanic realm (*n* = 57). The paralarvae grown in captivity showed high variability in size throughout their development, especially evident at days 15 and 25 (Table 2). One paralarva at day 20 was lost during the dissection and therefore, not included in the microbial analysis.

**Table 2:** *Octopus vulgaris* paralarvae analyzed in this work from NW Iberian Peninsula (CAIBEX-I), Morocco (CAIBEX-III), and aquaculture, showing the averaged dorsal mantle length (DML), sucker number, depth sampled (wild paralarvae), and distance to coast.

**Survey**	**Location**	***n***	**DML (mm)**	**Sucker *n*°**	**Depth (m)**	**Distance to coast (km)**
NW Iberian Peninsula	Coast	10	1.97 ± 0.43	3	62–136	10–15
	Ocean	31	2.59 ± 0.81	3–5	1,940–3,105	62–75
Morocco	Coast	9	2.02 ± 0.26	3	88–90	19
	Ocean	26	2.78 ± 0.59	3–15	787–3,110	48–171
Aquaculture	Day 0	5	1.42 ± 0.08	3	–	–
	Day 5	5	1.86 ± 0.29	3	–	–
	Day 10	5	2.25 ± 0.24	3	–	–
	Day 15	5	2.50 ± 0.49	3–5	–	–
	Day 20	4	2.10 ± 0.16	3–5	–	–
	Day 25	5	2.67 ± 0.44	3–5	–	–

### Sequence analysis

A total of 13,688,392 HTS reads were generated from the amplicon sequencing and 10,260,748 were retained after quality filtering. Kraken analysis revealed 0.023% and 0.007% of viral and archaeal sequences respectively, thus leaving 10,257,748 bacterial reads for further classification. The mean number of reads (± standard deviation) obtained per octopus sample was 96,406 ± 35,302 (range: 571–164,583) and 33,784 ± 18,495 in the zooplankton species (range: 9,840–50,974). Of these bacterial reads, 97.2% were successfully classified at phylum level (*n* = 28 phyla), 95.0% to class (*n* = 61), 93.4% to order (*n* = 123), 90.2% to family (*n* = 275), 83.7% to genus (*n* = 829), and 57.3% to species (*n* = 2,856) using the Greengenes taxonomy database. There was a consistent number of average reads, taxa identified and % of reads classified on the three types of octopus samples analyzed (aquaculture, NW Spain and Morocco) with no statistical differences among them (Table 3). However, the average number of reads and taxa identified were significantly lower in the zooplankton analyzed than in the octopus samples, but not the average % of reads classified (Table 3).

**Table 3:** Averaged number of reads (reads), taxa identified, and percentage of bacterial reads (%) classified to different taxonomic levels.

		**Aquaculture (*n* = 29)**	**NW Spain (*n* = 41)**	**Morocco (*n* = 35)**	**Zooplankton (*n* = 4)**
Phyla	Reads	81,955 ± 24,361	105,222 ± 34,435	89,882 ± 39,311	33,416 ± 18,229
	Taxa	19.59 ± 2.75	21.27 ± 2.55	20.80 ± 2.68	13.00 ± 1.63
	%	0.94 ± 0.05	0.98 ± 0.02	0.98 ± 0.01	0.99 ± 0.01
Class	Reads	79,347 ± 23,431	103,067 ± 33,631	88,147 ± 38,445	33,168 ± 18,095
	Taxa	33.79 ± 5.54	38.83 ± 5.24	38.26 ± 5.52	22.50 ± 2.08
	%	0.91 ± 0.05	0.96 ± 0.02	0.96 ± 0.02	0.98 ± 0.02
Order	Reads	78,192 ± 22,934	101,067 ± 32,824	86,875 ± 37,925	32,863 ± 17,802
	Taxa	72.17 ± 9.28	80.07 ± 10.29	79.20 ± 10.27	47.00 ± 4.90
	%	0.90 ± 0.05	0.94 ± 0.03	0.95 ± 0.03	0.98 ± 0.02
Family	Reads	76,090 ± 21,554	96,912 ± 31,615	84,180 ± 36,497	32,579 ± 17,776
	Taxa	154.86 ± 22.10	175.80 ± 27.26	174.46 ± 23.97	95.75 ± 14.17
	%	0.88 ± 0.05	0.91 ± 0.08	0.92 ± 0.04	0.97 ± 0.01
Genus	Reads	73,179 ± 19,904	89,748 ± 29,847	75,834 ± 33,424	31,912 ± 17,389
	Taxa	305.07 ± 72.02	390.61 ± 88.86	397.86 ± 72.89	162.75 ± 32.87
	%	0.85 ± 0.06	0.84 ± 0.08	0.82 ± 0.05	0.94 ± 0.01
Species	Reads	56,371 ± 16,415	58,778 ± 20,451	50,194 ± 21,556	20,296 ± 12,262
	Taxa	394.10 ± 141.04	583.80 ± 163.95	579.43 ± 151.08	267.25 ± 71.08
	%	0.66 ± 0.10	0.55 ± 0.07	0.55 ± 0.06	0.60 ± 0.12

A statistical relationship between the number of reads and the concentration of PCR product (ng/μl) after the purification step was obtained (Figure S1), whereby samples with <0.75 ng/μl prior to pooling showed a direct relationship between the initial concentration and reads obtained (*R*^2^ = 0.78). Interestingly, this relationship was not observed (*R*^2^ = 0.003) for those samples with >0.75 ng/μl that were diluted before pooling the samples, suggesting that the most consistent results were obtained by starting with a higher DNA concentration and diluting it to a standard concentration prior to amplicon sample preparation for high throughput sequencing.

### Microbial community structure and ontogenic changes

**Figure 2:**
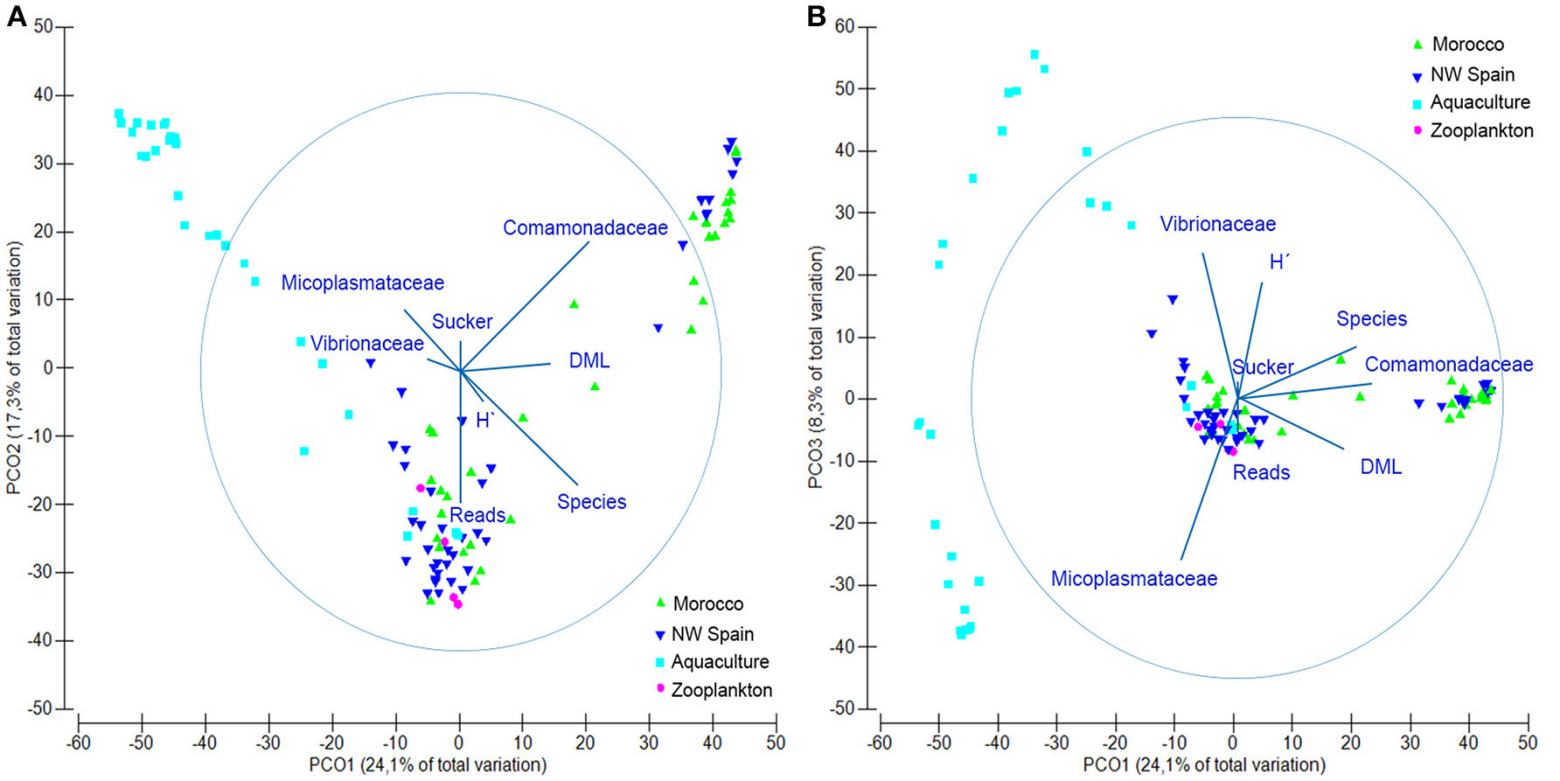
Principal coordinate analysis (PCO) plot showing the microbial communities found in *Octopus vulgaris* paralarvae collected in the wild (green) and reared in captivity (dark blue), as well as their zooplankton prey (light blue). **(A)** Axes PCO1 vs. PCO2 showing the main drivers (vectors) of variation in the microbial communities. **(B)** Axes PCO1 vs. PCO3. Overlaid variable vectors represent the strength of the correlations with the different PCO axes obtained with the distance linear model, being the circle considered as the unity. DML, dorsal mantle length; H′, Shannon's diversity index; Reads, total reads passing filter; Species, number of bacterial species; Sucker, number of suckers.

The microbial communities detected in *O. vulgaris* paralarvae collected in the wild were statistically different to microbiomes sampled from aquaculture paralarvae (PERMANOVA test, *p* = 0.001), with both communities pointing in opposite directions of the main axis of variation (Figure 2A). PCO1 accounted for 24.1% of the total variability detected in the resemblance matrix and was driven by the difference between captive (negative values) and wild paralarvae (positive values), and the DML of the paralarvae (thus showing ontogenic changes). PCO2 accounted for 17.3% of the total variation and was primarily driven by the number of species detected and number of reads, with positive/negative values indicating fewer/higher number of species and reads. PCO3 accounted for 8.5% of total variability and was driven by the two dominating bacterial families identified from the paralarvae in aquaculture, with positive values showing the samples dominated by Vibrionaceae and negative values Mycoplasmataceae (Figure 2B). In summary, wild paralarvae had on average more bacterial species and diversity than paralarvae reared in aquaculture and zooplankton (Table 3), while the percentage of reads identified was higher in captive paralarvae.

**Figure 3:**
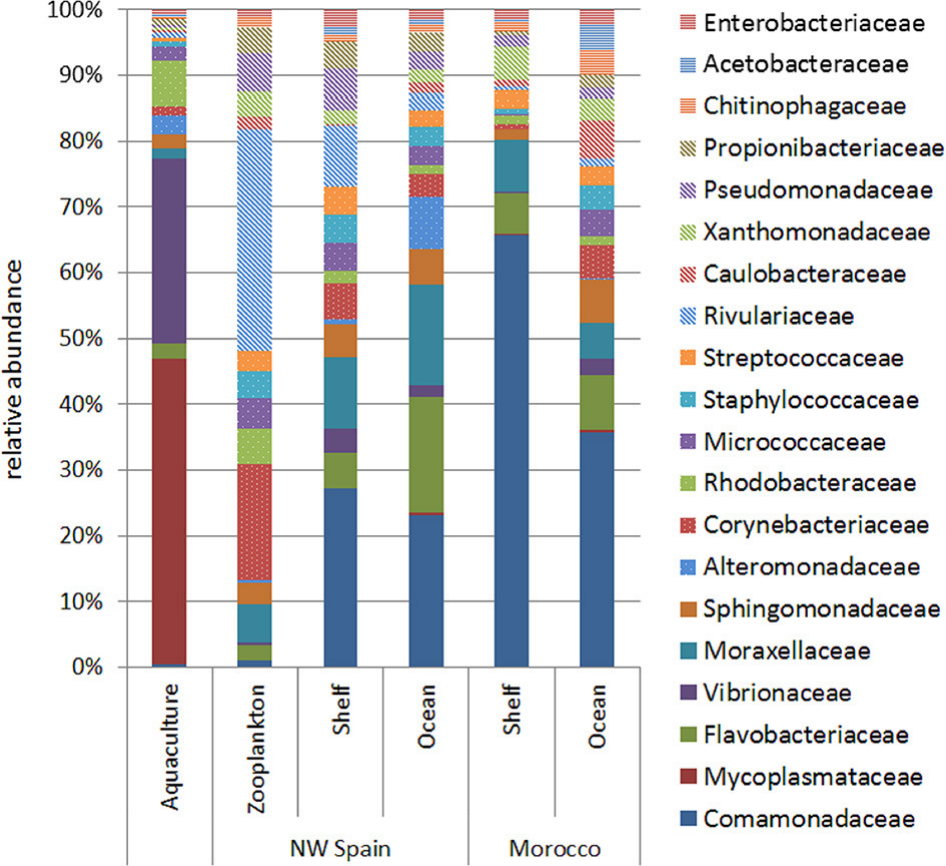
Relative abundance of the main bacterial families (representing more than 1% of total reads) detected in the digestive tract of the zooplankton collected over the shelf (zooplankton) and in *Octopus vulgaris* paralarvae reared in captivity (aquaculture) and collected in the wild (NW Spain and Morocco). Wild paralarvae were classified according to their location, with samples collected over the continental shelf (<200 m) named as “shelf,” and those collected over the continental slope (>200 m) named as “ocean.”

Analysis of bacterial families within each of the sample groups studied revealed qualitative differences between aquaculture and wild paralarvae/zooplankton groups (Figure 3). The bacterial families detected in the zooplankton differed to that of the paralarvae collected over the continental shelf of NW Spain, especially the families Corynebacteriaceae and Rivulariaceae, which were more abundant in the zooplankton. In addition, the microbial communities of wild paralarvae from both upwelling systems clearly differed depending on the location where the paralarvae were collected (shelf vs. ocean, Figure 3). These differences were consistent in both upwelling systems, NW Spain and Morocco (PERMANOVA test, *p* = 0.011), with families more evenly distributed on average in the ocean than the shelf regions.

**Figure 4:**
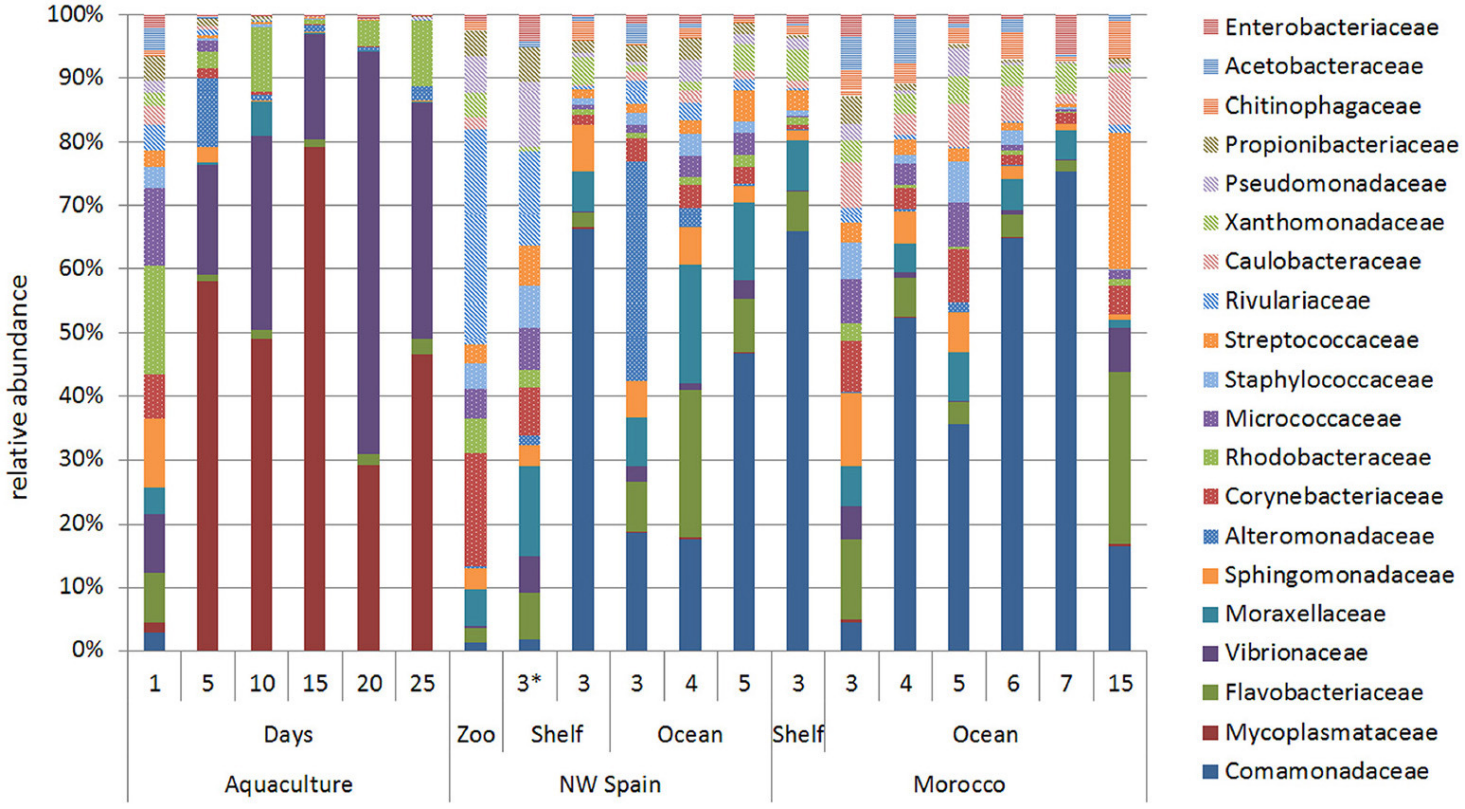
Relative abundance of the main bacterial families showing the ontogenic changes in the microbial communities of *Octopus vulgaris* paralarvae reared in captivity for 25 days and in two upwelling regions of the North Eastern Atlantic: NW Spain and Morocco. The numbers present in the two upwelling regions represent the sucker number. The microbial community found in the zooplankton prey (Zoo) is represented together with the paralarvae collected in the same coastal sample (asterisk).

The ontogenic changes in the microbial community were evident when the age of the paralarvae, measured as days in captivity and sucker number in the wild, was taken into account (Figure 4). Surprisingly, paralarvae hatched in captivity (day 0) had a diverse microbial flora (averaged number of species ± standard deviation, 636 ± 50), which was not significantly different (PERMANOVA test, *p* = 0.052) from that of recently hatched paralarvae in the wild (619 ± 189, marked with an asterisk in Figure 4). However, the microbial diversity recorded in aquaculture at day 0 was significantly higher (PERMANOVA test, *p* = 0.001) than the rest of the samples collected in aquaculture, with averages of 360 ± 149 (day 5), 321 ± 100 (day 10), 288 ± 62 (day 15), 367 ± 31 (day 20), and 385 ± 64 (day 25) bacterial species. The families Mycoplasmataceae and Vibrionaceae dominated the microbial communities of captive paralarvae from day 5 (68%) onwards, accounting for more than 82% of the total reads at day 25 (Figure 4).

The opposite trend was observed in the wild paralarvae, where the bacterial richness gradually increased to a maximum of 919 and 801 species in NW Spain and Morocco, respectively. Paralarvae caught close to the shore were found to have an even representation of bacterial families, similar to that of the zooplankton, whereas the GI of samples collected away from the shore (>4 suckers) were enriched with species of the family Comamonadaceae in both upwelling systems (Figure 4). The same interpretation can be drawn from the direction of the vectors DML and Comamonadaceae (Figure 2), pointing toward the oldest paralarvae in the wild, thus showing that the main differences in the oceanic paralarvae were due to an increase in size (DML) and an incorporation of bacterial species of the family Comamonadaceae.

**Figure 5:**
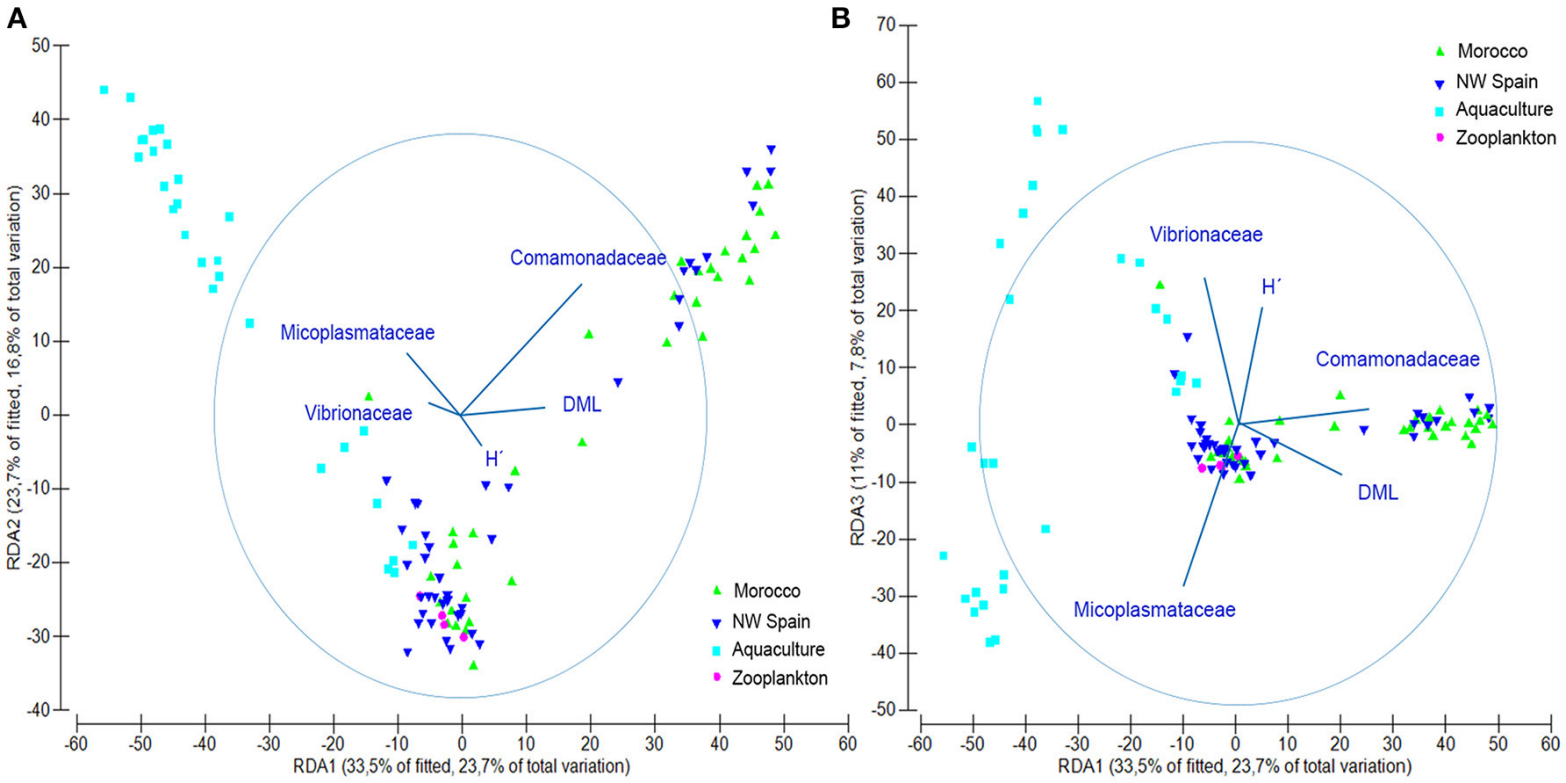
Redundancy analysis (RDA) plot showing the output of the fitted distance linear model obtained with only 5 variables, accurately representing the microbial community structure found in this study. **(A)** RDA1 vs. RDA2 axes and **(B)** RDA1 vs. RDA3 axes. Abbreviations defined in Figure 2.

DistLM results showed that the examined variables accounted for 31.68% (*Octopus*), 28.12% (*Experiment*), 26.88% (*Taxonomy*), and 15.28% (*Run*) of the total variability found in the microbial communities. When considered altogether, they accounted for up to 58.88% of the total microbial variability as follows: 31.68% (*Octopus*) + 15.76% (*Taxonomy*) + 6.85% (*Run*) + 4.59% (*Experiment*). The simplest model that accurately reproduces the microbial community structure obtained in this study (Figure 5), included five variables accounting for up to 50.4% of total variability: 21.42% (Comamonadaceae) + 15.75% (Mycoplasmataceae) + 9.28% (Vibrionaceae) + 2.32% (DML) + 1.62% (H′). This simple model reproduces both the variability found in the different samples analyzed as well as the ontogenic changes in bacterial communities. The contribution of the different variables to the different dbRDA axes showed that Comamonadaceae were characteristic of wild paralarvae, whereas Mycoplasmataceae and Vibrionaceae were largely found in captive paralarvae. This reduced model also highlighted the importance of *Octopus* DML and bacterial diversity (H′), since bacterial diversity was differentially correlated with size of the paralarvae between the wild and captive samples.

### Core gut microflora

RELATE analyses revealed the main bacterial families driving both the similarities (Table 4) and the differences (Table 5) between the sample groups. Since paralarvae hatched in captivity (day 0) had a similar microbial community to wild paralarvae (Figure 4), we combined this group with the wild paralarvae to infer the “core” gut microbiota of healthy *Octopus* paralarvae (i.e., the common families to all paralarvae that declined in captivity). The importance of the families Flavobacteriaceae, Comamonadaceae, Moraxellaceae, and Sphingomonadaceae was evident in the wild paralarvae, with their contributions changing from one upwelling region to the other (Table 4). These differences are consistent with the statistical differences revealed by the PERMANOVA analysis among both upwelling systems, despite the main families being largely the same (Figure 4). In the zooplankton, the main bacterial family was Corynebacteriaceae which contributes up to 27.81% of the species present; in contrast, this family only represented between 2.89 and 5.16% of the bacteria found in the wild paralarvae collected in both upwelling systems and 0.85% in aquaculture (Figure 4).

**Table 4:** Top 10 most discriminant bacterial families of the different *Octopus vulgaris* paralarvae analyzed and their zooplankton prey.

**Sample**	**Similarity**	**Families**	**Av. ab**.	**Con%**	**Cumulative**
NW Spain	31.59	Flavobacteriaceae	0.10	16.99	16.99
		Comamonadaceae	0.13	15.49	32.49
		Moraxellaceae	0.08	13.55	46.04
		Sphingomonadaceae	0.03	6.11	52.15
		Corynebacteriaceae	0.03	5.16	57.31
		Staphylococcaceae	0.02	4.04	61.35
		Propionibacteriaceae	0.02	3.96	65.31
		Micrococcaceae	0.02	3.81	69.13
		Rivulariaceae	0.03	3.43	72.56
		Streptococcaceae	0.02	3.42	75.98
Morocco	42.49	Comamonadaceae	0.26	41.07	41.07
		Moraxellaceae	0.04	9.21	50.28
		Flavobacteriaceae	0.05	8.37	58.65
		Sphingomonadaceae	0.03	5.33	63.98
		Caulobacteraceae	0.03	3.91	67.90
		Xanthomonadaceae	0.03	3.85	71.75
		Chitiniphagaceae	0.02	2.90	74.65
		Corynebacteriaceae	0.02	2.89	77.54
		Bradyrhizobiaceae	0.02	2.12	79.65
		Streptococcaceae	0.02	2.08	81.73
Aquaculture	38.12	Mycoplasmataceae	0.31	52.56	52.64
		Vibrionaceae	0.20	29.60	82.15
		Rhodobacteraceae	0.05	5.67	87.83
		Flavobacteriaceae	0.02	2.70	90.53
		Alteromonadaceae	0.02	1.47	92.00
		Corynebacteriaceae	0.01	0.85	92.84
		Sphingomonadaceae	0.01	0.79	93.64
		Moraxellaceae	0.01	0.70	94.33
		Enterobacteriaceae	0.00	0.70	95.03
		Micrococcaceae	0.01	0.67	95.70
Zooplankton	37.72	Corynebacteriaceae	0.11	27.81	27.81
		Moraxellaceae	0.04	10.89	38.70
		Pseudomonadaceae	0.05	10.50	49.20
		Micrococcaceae	0.05	8.47	57.68
		Staphylococcaceae	0.03	7.05	64.73
		Propionibacteriaceae	0.03	6.44	71.17
		Sphingomonadaceae	0.03	6.34	77.51
		Streptococcaceae	0.04	3.89	81.40
		Rivulariaceae	0.14	3.62	85.02
		Microbacteriaceae	0.01	2.69	87.71

*Averaged abundance (Av. ab.), contribution percentage to the total variability (Con%) and the cumulative variability explained by the families*.

**Table 5:** Top 10 most discriminative bacterial families driving the differences between the groups studied, with the averaged abundances (Ab.) represented for each group and their contribution percentage to the total variability (Con%).

**Contrast**	**Dissimilarity**	**Families**	**Ab. NW**	**Ab. Mo**.	**Con%**	**Cumulative**
NW Spain vs. Morocco	66.63	Comamonadaceae	0.13	0.26	25.78	25.78
		Flavobacteriaceae	0.10	0.05	9.30	35.07
		Moraxellaceae	0.08	0.04	7.34	42.41
		Vibrionaceae	0.02	0.03	4.23	46.65
		Sphingomonadaceae	0.03	0.03	3.78	50.43
		Alteromonadaceae	0.03	0.00	3.39	53.81
		Corynebacteriaceae	0.03	0.02	2.96	56.77
		Caulobacteraceae	0.01	0.03	2.85	59.62
		Micrococcaceae	0.02	0.02	2.79	62.41
		Pseudomonadaceae	0.02	0.01	2.78	65.18
			**Ab. Wild**	**Ab. Aq**.		
Wild vs. Aquaculture	90.87	Mycoplasmataceae	0.26	0.37	28.11	28.11
		Vibrionaceae	0.04	0.24	17.43	45.54
		Comamonadaceae	0.05	0.00	12.99	58.53
		Flavobacteriaceae	0.03	0.01	4.97	63.51
		Moraxellaceae	0.03	0.01	4.76	68.27
		Rhodobacteraceae	0.03	0.04	3.56	71.83
		Alteromonadaceae	0.02	0.02	2.75	74.58
		Sphingomonadaceae	0.02	0.00	2.59	77.17
		Micrococcaceae	0.02	0.00	1.87	79.04
		Corynebacteriaceae	0.02	0.00	1.86	80.91
			**Ab. Wild**	**Ab. Zoo**.		
Wild vs. Zooplankton	72.78	Comamonadaceae	0.19	0.01	17.46	17.46
		Rivulariaceae	0.02	0.14	14.30	32.76
		Corynebacteriaceae	0.02	0.11	9.32	41.08
		Flavobacteriaceae	0.08	0.01	6.62	47.70
		Moraxellaceae	0.06	0.04	4.97	52.67
		Pseudomonadaceae	0.02	0.05	4.93	57.61
		Micrococcaceae	0.02	0.05	4.25	61.86
		Streptococcaceae	0.02	0.04	3.85	65.71
		Xanthomonadaceae	0.02	0.03	3.22	68.93
		Rhodobacteraceae	0.01	0.03	3.03	71.95
			**Ab. Aq**.	**Ab. Zoo**.		
Aquaculture vs. Zooplankton	88.23	Mycoplasmataceae	0.1	0.00	24.41	24.41
		Vibrionaceae	0.20	0.00	16.00	40.41
		Rivulariaceae	0.00	0.14	11.66	52.07
		Corynebacteriaceae	0.01	0.11	8.47	60.54
		Rhodobacteraceae	0.05	0.03	4.50	65.04
		Pseudomonadaceae	0.00	0.05	4.08	69.12
		Micrococcaceae	0.01	0.05	4.02	73.13
		Moraxellaceae	0.01	0.04	3.56	76.70
		Streptococcaceae	0.00	0.04	3.06	79.76
		Xanthomonadaceae	0.00	0.03	2.29	82.04

Of the main families contributing to the dissimilarities between the groups analyzed (Table 5), the Family Comamonadaceae was determinant in the differentiation of all *Octopus* groups; this family was abundant in the paralarvae collected off Morocco but nearly absent in the paralarvae grown in captivity. These captive paralarvae were characterized by Mycoplasmataceae and Vibrionaceae families, while Rivulariaceae and Corynebacteriaceae were the main discriminant families of the zooplankton prey.

## Discussion

In this study, we present the first analysis of the GI microbiome of *O. vulgaris* paralarvae, characterizing both the complex microbial communities present in wild paralarvae and the ontogenic change in bacterial community composition based on diet and development in captivity. Paralarvae reared in captivity with *Artemia* showed a depletion of bacterial diversity, particularly after day 5 when almost half of the bacterial species present at day 0 were lost. In contrast, bacterial diversity increased in wild paralarvae as they developed in the ocean (Figure 4), likely due to the exposure of new bacterial communities via ingestion of a wide diversity of prey (Roura et al., 2012; Olmos-Pérez et al., 2017).

The number of bacterial sequences obtained per sample (average of 96,406 ± 35,302 *SD*) was almost 10 times the minimum sample depth needed to capture the structure of microbial communities (Caporaso et al., 2011). Only two samples had <10,000 sequences (833 and 8,574) and, despite their low depth, the main bacterial groups and their relative proportions were consistent with other samples. Despite using Greengenes, the most comprehensive microbial taxonomy database available (McDonald et al., 2012), we found a high percentage of unclassified sequences that may represent novel bacterial species present (between 20 and 60% per sample). High proportions of unclassified sequences have been described in other studies including mouse gut and anaerobic digester samples, where phylotypes unclassified at the genus level represented a greater proportion of the total community variation than classified OTUs, underscoring the need for greater diversity in existing reference databases (Werner et al., 2012). In our study, the percentage of unclassified reads explained up to 7.2% of the total variability found in the microbial communities. Interestingly, these unclassified OTUs were significantly more abundant in wild than captive paralarvae, indicating a high degree of novelty in the microbial species present in the digestive tract of wild paralarvae incorporated through the diet.

Consistent with this study, previous genomic studies have found that wild fish larvae have more diverse microflora than their captive relatives (e.g., Atlantic cod: Dhanasiri et al., 2011; olive flounder: Kim and Kim, 2013). This suggests that monospecific (*Artemia*) or even formulated diets are not as favorable as those diets encountered in nature, which seem to provide an important source of potentially beneficial microorganisms that might be exploited to supplement and diversify depleted microflora in captivity. The intestinal microbiota of a host can be classified as autochthonous (i.e., core bacteria in this study) or allochthonous bacteria (Ringø and Birkbeck, 1999). The autochthonous bacteria are those able to colonize the host's gut epithelial surface (microvilli), while the allochthonous bacteria are transient, associated with food or water, and cannot colonize except under abnormal conditions. Several studies have demonstrated that the endogenous microbiota is an important component of the mucosal barrier, representing the first line of defense against pathogens (Gómez and Balcázar, 2008). The diverse core bacteria (autochthonous) detected in recently hatched *O. vulgaris* and wild paralarvae was rapidly modified and substituted by two opportunistic bacterial families, Vibrionaceae followed by Mycoplasmataceae (Figure 4). The same succession of opportunistic bacteria was also detected in cod larvae reared in captivity (McIntosh et al., 2008). Both families are known pathogens affecting many larviculture systems, with the family Vibrionaceae often found in parasitic or mutualistic associations with the gut of marine animals, where they provide diverse metabolic capabilities (Thompson et al., 2004; Sullam et al., 2012; Zhao et al., 2012).

Although certain *Vibrio* species are beneficial for the host (Austin et al., 2005; Fjellheim et al., 2007), this opportunistic group is responsible for high mortalities in larviculture (Brunvold et al., 2007; Zhao et al., 2012). Indeed, studies have shown that *Artemia* are important vectors of pathogens (mostly Vibrionaceae) that colonize fish (e.g., McIntosh et al., 2008; Reid et al., 2009) and abalone larvae after first feeding (Zhao et al., 2012). Interestingly, Vibrionaceae was identified using a culture-dependent method and 16S rDNA clone library in wild adult specimens of *O. mimus*, however most of the cloned sequences belonged to the family Mycoplasmataceae (Iehata et al., 2015). They suggested that *Mycoplasma* might be a autochthonous member of the octopus GI bacterial community with an unknown function, as is has also been found within the GI tract of wild specimens of Norway lobster (Meziti et al., 2010) and Atlantic salmon (Star et al., 2013). Our results indicated that this genus is present in both, wild and captive paralarvae, but their abundance is markedly different (Table 5). However, we suggest that the *Mycoplasma* species observed in captive *Octopus* paralarvae are opportunistic and, together with *Vibrio*, are candidate pathogens that may be responsible for the high mortalities observed in *Octopus* larviculture. *Mycoplasma* has also been detected in farmed salmon sporadically, but when present, it dominated the GI tract communities (Zarkasi et al., 2014). The sporadic nature of *Mycoplasma* suggests host factors at play that may influence GI tract community structure and contribute to dynamic changes. The saprophytic nature of *Mycoplasma*, with a fermentative metabolism, and its increasing abundance in captive octopus paralarvae may be related with the presence of dead paralarvae and *Artemia* at the bottom of the tank, which provide optimal conditions for this opportunistic genus. More research is needed to accurately identify the different *Mycoplasma* and *Vibrio* strains in order to test this hypothesis.

In our study, the diversity of the GI microbiota found in recently hatched paralarvae in captivity (day 0) was unexpectedly high (Figure 4). Olafsen (2001) suggested that a dense, diverse but non-pathogenic egg epiflora may be a barrier against colony formation by pathogens. One possible explanation is that the diverse microbiota in captive hatchlings of *Octopus* might be derived from bacteria attached to the egg capsule. This suggestion is supported by the observed biodiversity of culturable epiflora associated with healthy eggs of *O. mimus* (Iehata et al., 2016). Bacterial diversity of healthy eggs was higher than that of infected eggs (i.e., eggs from the same female that changed color from whitish to yellow-brownish indicative of infection), which were dominated by pathogenic genera like *Pseudoalteromonas, Vibrio*, and *Tenacibaculum*. In our study, the initial diversity rapidly decreased when *Octopus* paralarvae started exogenous feeding on *Artemia*, and opportunistic bacteria colonized the GI tract. In contrast, a gradual increase in species richness was observed among wild paralarvae as they migrated from their coastal hatchling grounds to the oceanic realm (Figures 3, 4). This is the first time that this ontogenic change has been observed in *O. vulgaris* paralarvae and suggests a relationship between diversity of GI flora and paralarvae survival. This ontogenic change in the microbial community has also been observed in other marine organisms, including abalone (Zhao et al., 2012), sponges (Cao et al., 2012), white shrimp (Huang et al., 2016) and fish aquaculture (reviewed in Ringø et al., 2016), and has been suggested to be a natural process that likely plays a role in the correct development of the host's immune system and GI tract, preventing pathogens from colonization.

Marine larvae are in constant interaction with bacteria during their first feeding (Olafsen, 2001), and compared to wild conditions, intensively cultured larvae experience stress due to inappropriate feeding (Iglesias et al., 2007) and higher larval densities than in their oceanic environment (Roura et al., 2016). Furthermore, the high organic load associated with rearing conditions may enhance the proliferation of opportunistic pathogenic bacteria (Lauzon et al., 2010), which can be detrimental to the paralarvae and is one potential cause for the highly unpredictable growth and reduced survival that limits *Octopus* aquaculture. Our results clearly demonstrate that the gut flora of captive paralarvae was distinctly different from the “healthy” gut flora community of wild paralarvae (Figures 3, 4).

The bacterial families Comamonadaceae, Flavobacteriaceae, and Moraxellaceae were the most discriminating families enriched in the wild *Octopus* paralarvae core community, and could be a potential source of beneficial bacteria to test in captivity. This was the case of wild olive flounder (Kim and Kim, 2013), where wild fishes were an essential source of beneficial microbes that conferred resistance to pathogenic bacteria (Nayak, 2010). Bacterial composition in wild *O. vulgaris* (at the phylum level) was similar to carnivorous/herbivorous marine fishes (Sullam et al., 2012), with a composition hierarchy consisting of Proteobacteria>Actinobacteria>Bacteroidetes>Firmicutes. Interestingly, one of the core intestinal bacterial groups of wild *Octopus* paralarvae was the family Flavobacteriaceae (Table 4). Although this family was initially proposed to be exclusively found in herbivorous fishes (Sullam et al., 2012), this hypothesis was later rejected by a pyrosequencing study that found this group within the GI tract of wild Atlantic cod (Star et al., 2013).

Finally, it is remarkable the similarity of the microbial community found in wild zooplankton and that of the paralarvae growing near the coast (Figure 4). Although only four zooplankton species were analyzed in this study, the similarities observed support a close relationship between the microbial communities present in GI tract of the predator and that of its prey. Wild paralarvae continuously diversify their core gut microflora with a diverse diet (Roura et al., 2012; Olmos-Pérez et al., 2017), which provides a natural source of allochthonous bacteria. This diverse microbiota likely serve a variety of functions in the nutrition and health of the host by promoting nutrient supply, preventing the colonization of infectious agents, energy homeostasis and maintenance of normal mucosal immunity (Nayak, 2010). In summary, this study provides a comprehensive overview of the bacterial communities inhabiting the GI tract of *O. vulgaris* paralarvae, and reveals new research lines to challenge the current bottlenecks preventing sustainable octopus aquaculture.

## Author contributions

AR, JS, and SD planned the work. AR captured the wild paralarvae and MN grew the paralarvae in captivity. AR, JS, and SD designed the genomic study. AR and SD performed the genomic analyses. AR, SD, JS, and MN wrote the manuscript. All authors have read and approved the content of the manuscript.

### Conflict of interest statement

The authors declare that the research was conducted in the absence of any commercial or financial relationships that could be construed as a potential conflict of interest.

## Supplementary material

Figure S1 Effect on the number of reads obtained per sample depending on the initial concentration of the PCR product after the cleaning step. PCR products with concentration below/above 0.5 ng/μL are shown in red/blue, respectively.
