# Supplementary figures and images for: You Are What You Eat: A Genomic Analysis of the Gut Microbiome of Captive and Wild Octopus vulgaris Paralarvae and Their Zooplankton Prey
Source: Front Physiol. 2017 May 31;8:362. doi: 10.3389/fphys.2017.00362 (PMC5450036; doi:10.3389/fphys.2017.00362)

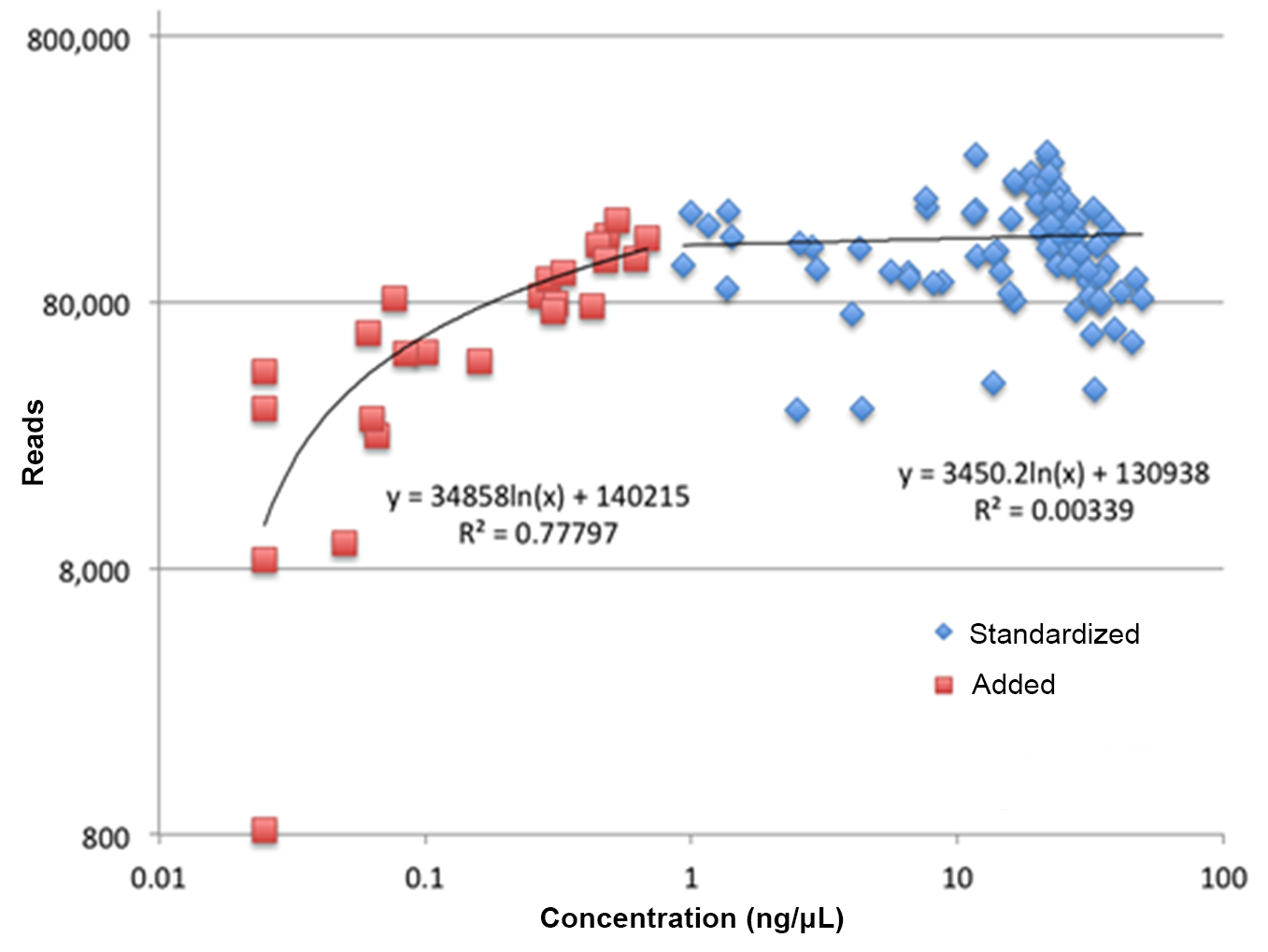

Supplement: Figure S1 — Effect on the number of reads obtained per sample depending on the initial concentration of the PCR product after the cleaning step. PCR products with concentration below/above 0.5 ng/μL are shown in red/blue, respectively. [file Image1.TIF]
